# Sensitivity of Serum Beta-D-Glucan in Candidemia According to *Candida* Species Epidemiology in Critically Ill Patients Admitted to the Intensive Care Unit

**DOI:** 10.3390/jof8090921

**Published:** 2022-08-30

**Authors:** Malgorzata Mikulska, Laura Magnasco, Alessio Signori, Chiara Sepulcri, Silvia Dettori, Stefania Tutino, Antonio Vena, Franca Miletich, Nadir Ullah, Paola Morici, Lorenzo Ball, Paolo Pelosi, Anna Marchese, Daniele Roberto Giacobbe, Matteo Bassetti

**Affiliations:** 1 Department of Health Sciences (DISSAL), University of Genoa, 16132 Genoa, Italy; 2 Division of Infectious Diseases, San Martino Policlinico Hospital, IRCCS for Oncology and Neurosciences, 16132 Genoa, Italy; 3 Department of Health Sciences (DISSAL), Section of Biostatistics, University of Genoa, 16132 Genoa, Italy; 4 Microbiology Unit, San Martino Policlinico Hospital, IRCCS for Oncology and Neurosciences, 16132 Genoa, Italy; 5 Department of Surgical Sciences and Integrated Diagnostics (DISC), University of Genoa, 16132 Genoa, Italy; 6 Anesthesia and Critical Care, San Martino Policlinico Hospital, IRCCS for Oncology and Neurosciences, 16132 Genoa, Italy

**Keywords:** ICU, sensitivity, BDG, *Candida*

## Abstract

Serum beta-D-glucan (BDG) determination plays an important role in the diagnosis of candidemia among critically ill patients admitted to the intensive care unit (ICU). However, BDG levels measured may be lower in the case of infections caused by some non-*albicans* species, such as *C. parapsilosis* and *C. auris*. The aim of this single-center study was to investigate the sensitivity of serum BDG for the diagnosis of candidemia stratified according to causative *Candida* species in ICU patients. This was a single-center, retrospective study, including all adult patients admitted to ICU during the period 2018–2021. All episodes of candidemia with a determination of BDG available within 3 days before or after positive blood culture were recorded. The preplanned primary objective was to investigate the sensitivity of serum BDG to detect candidemia early and the effect of different *Candida* species. The secondary objective was to measure serum BDG in patients with candidemia from different *Candida* species. In total, 146 candidemia episodes in 118 patients were analyzed. Median BDG value for *C. albicans* candidemia (182 pg/mL) was higher than that observed for *C. parapsilosis* (78 pg/mL, *p* = 0.015) and *C. auris* (48 pg/mL, *p* = 0.022). The overall sensitivity of BDG for the diagnosis of candidemia was low (47%, 95% CI 39–55%). In conclusion, in critically ill patients admitted to ICU, serum BDG levels for candidemia were different among species, with lower levels confirmed for *C. parapsilosis* and *C. auris*. Serum BDG sensitivity for early detection of candidemia was lower than previously reported in other ICU populations.

## 1. Introduction

1,3-beta-D-glucan (BDG) is a key component of the cell wall of fungi, and its determination in serum samples is an indirect microbiological tool used to support the diagnosis of invasive fungal diseases [1].

Serum BDG has different sensitivity and specificity according to the characteristics of the baseline patient population considered [2]. Among critically ill patients admitted to the intensive care unit (ICU) and non-hematological patient population, the main limitation of serum BDG to detect candidemia has been a low specificity (with acceptable sensitivity) [3,4], while low sensitivity (but high specificity) has been reported in patients with hematological malignancies [5,6]. In the ICU, even with reported rather good sensitivity and specificity (respectively, 86% and 71%), low positive predictive value (PPV) has been reported at low prevalences of candidemia, with high negative predictive value (NPV) [3,7]. The use of a higher than standard cut-off for positivity has been suggested as potentially useful in ICU patients to increase PPV [8]. Despite these concerns, BDG has been supported for early diagnosis of invasive fungal infections and pre-emptive therapy in critically ill patients admitted to the ICU by international guidelines [9,10]. In a recently published study, good NPV and sensitivity were described among critically ill patients [11]. Although the evaluation of such parameters was not the primary aim of the study, the authors concluded that a negative value of serum BDG might be used as the only tool to discontinue empirical antifungal therapy in the ICU. However, serum BDG values might also be influenced by the fungal species responsible for the infection. A lower sensitivity of serum BDG in *C. parapsilosis* candidemia has been previously described [11], and growing evidence suggests a similar lower sensitivity in the detection of *C. auris* invasive infection [12,13,14]. Accordingly, we hypothesized that, in ICU patients, BDG sensitivity to detect candidemia early could be different for different *Candida* species. Consequently, the major aim of the present study was to assess the sensitivity of serum BDG in a population of critically ill patients diagnosed with candidemia and to evaluate possible differences in sensitivity according to the *Candida* species responsible for the infection.

## 2. Materials and Methods

### 2.1. Study Design and Patient Selection

This is a single-center, retrospective study conducted in the ICUs in Policlinico San Martino Hospital, Genoa, Italy, in the period between 1 January 2018 and 30 September 2021. Inclusion criteria for the present study were: (i) age ≥18 years, (ii) admission to any of the ICUs in our hospital, (iii) at least one blood culture positive for *Candida* spp., and (iv) a value of serum BDG available within 3 days before or after positive blood culture collection. In the case of multiple episodes of candidemia caused by the same species in the same patient during the study period, a new episode was considered only when at least 30 days elapsed after the last positive culture of the previous one. During the whole study period, four ICUs served our hospital, and a fifth was added during the novel coronavirus-19 disease (COVID-19) pandemic. The preplanned primary objective was to investigate the sensitivity of serum BDG to detect candidemia early and the effect of different *Candida* species on BDG sensitivity. The secondary objective was to descriptively compare serum BDG levels in patients with candidemia caused by different *Candida* species.

### 2.2. Microbiology

Blood cultures and BDG sampling were performed according to the caring clinicians’ judgment. Blood samples were cultured following standard recommendations by the automated Bactec method with both aerobic and anaerobic media (Bactec FX; Becton-Dickinson Microbiology Systems, Franklin Lakes, NJ, USA). *Candida* species identification in blood cultures was performed with matrix-assisted laser desorption ionization–time of flight mass spectrometry (MALDI-TOF MS—VITEK MS; bioMérieux, Marcy-l’Etoile, France), using VITEK MS v4.0 software. BDG levels were tested with Fungitell assay (Associates of Cape Cod, Falmouth, MA, USA), according to the manufacturer’s instructions. The established cut-off for positivity was 80 pg/mL.

### 2.3. Statistical Analysis

Categorical variables are expressed as an absolute number, percentage, and 95% confidence interval (95% CI) and confronted with the Chi-square test. Continuous variables are expressed as median values and interquartile range (IQR) or mean values ± standard deviation (SD). Serum BDG values were compared among the *Candida* species using the Kruskal–Wallis test, while the Student’s t-test was used to compare the mean times of BDG sampling. Statistical analyses were carried out with Stata (v.16; StataCorp; Collage Station, TX, USA).

### 2.4. Ethical Considerations

This study was performed in accordance with the guidelines of the Declaration of Helsinki. It was approved by the Liguria Ethics Committee (approval number 43/2022).

## 3. Results

**Figure 1:**
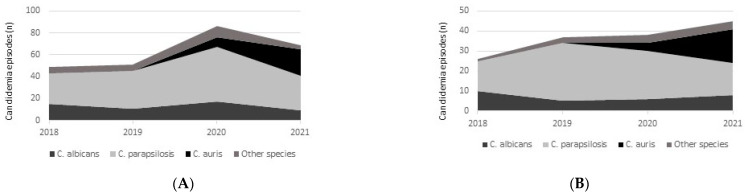
Distribution of *Candida* species responsible for candidemia over time, both in the overall ICU population (**A**) and among included patients (**B**).

During the study period, 255 episodes of candidemia occurred in patients admitted to the ICUs. Overall, 146 candidemia (57.3%) episodes of candidemia occurring in 118 critically ill patients met the inclusion criteria of concomitant BDG testing and were included in the present study. In total, 72 patients (61.0%) were males, and the median age of the cohort was 66 years (IQR 60–74 years). The representativity of the included candidemia episodes with regard to all the episodes occurring during the study period was similar for each *Candida* species considered: 29/52 episodes for *C. albicans* (55.8%), 84/144 episodes for *C. parapsilosis* (58.3%), 21/33 episodes for *C. auris* (63.6%) and 12/26 episodes for other species (46.2%). Change in species epidemiology over time is shown in Figure 1, both for the overall population of the ICU (Figure 1A) and for patients included in the present study (Figure 1B). Serum BDG levels and sensitivity are summarized in Table 1. Median serum BDG values were higher in the case of *C. albicans* compared to *C. auris* (*p* = 0.022) and to *C. parapsilosis* (*p* = 0.015), while no difference in BDG levels was present between *C. auris* and *C. parapsilosis* (*p* = 0.57). No statistically significant difference was observed in serum BDG sensitivity among different species: neither *C. albicans* vs. *C. auris* (*p* = 0.18) nor *C. albicans* vs. *C. parapsilosis* (*p* = 0.09). When restricting the analysis only to BDG values available after the first positive blood culture, overall sensitivity was 52.5% (95% CI 39.6–65.1%), while the sensitivity according to species was 71.4% (95% CI 41.1–90.0%) for *C. albicans*, 14.3% (95% CI 1.2–70.1%) for *C. auris*, 51.5% (95% CI 34.3–68.4%) for *C. parapsilosis*, and 60% (95% CI 10.6–95.0%) for other *Candida* species.

Among 37 candidemia episodes, 2 or more serum BDG values were available, and a description of concordance or discordance among values per episode is reported in Table 2. The mean difference observed between the lowest and the highest BDG value available per episode was 79.1 pg/mL (median 33 pg/mL, IQR 9–108 pg/mL). When stratifying these results according to *Candida* species, a different concordance between serial BDG samples was noted. Indeed, the observed median difference of BDG values for *C. albicans* episodes (*n* = 10) was 13 pg/mL (IQR 0–60 pg/mL), 86 pg/mL (IQR 39–91 pg/mL) for *C. auris* episodes (*n* = 5), and of 87.4 pg/mL (IQR 9–124 pg/mL) for *C. parapsilosis* episodes (*n* = 18). An increase over time in the values of serum BDG available for each episode was noted in 4 episodes (40%) of *C. albicans* candidemia, 6 episodes (33%) of *C. parapsilosis* candidemia, and 2 episodes (40%) of *C. auris* candidemia (see Supplementary Figure S1 for more details).

## 4. Discussion

In critically ill patients admitted to the ICU, serum BDG values in temporal proximity of *C. albicans* candidemia were higher when compared either with *C. parapsilosis* or *C. auris* candidemia. Serum BDG sensitivity for early detection of candidemia in our study was lower than previously reported in ICU populations.

A lower release of serum BDG in the case of *C. parapsilosis* [11] or *C. auris* [12,13] candidemia has been previously reported. While we did not observe a statistically significant difference in sensitivity of a positive BDG value for the diagnosis of candidemia sustained by different species (although it was numerically higher for *C. albicans*), a general consideration stemming from our population of critically ill patients is that the sensitivity of BDG was generally low, ranging from 40% to 60%.

The sensitivity observed in the present study is lower than the 81% (95% CI 74 to 86%) sensitivity reported in a recent meta-analysis including 10 studies for ICU patients at risk for candidemia and candidiasis [15,16]. However, stratification of serum BDG sensitivity by *Candida* species was not performed in previous studies. The most recent randomized clinical trial on the use of serum BDG for guiding antifungal therapy in critically ill patients [7] reported a sensitivity of 64.3% for candidemia, despite *C. albicans* being the most frequently isolated *Candida* species responsible for infection. The data on sensitivity based on species distribution have not been reported in previous studies. A recent randomized trial reported 100% sensitivity, but it was not designed to specifically investigate this aspect, carrying the limitation of the low sample size for this question and that only 33% (i.e., 2 of 6 episodes) of documented infections of the bloodstream were caused by non-*albicans* species [17]. Our study showed that lower absolute serum BDG levels were observed in candidemia caused by *C. auris*, followed by *C. parapsilosis*, and higher values were observed for *C. albicans* [11,12,13,14]. High variability was observed in serum BDG values in candidemia episodes sustained by non-*albicans* species. Overall, the low sensitivity of serum BDG for the diagnosis of candidemia and the high variability of BDG values might be explained by the pathogenesis of candidemia in patients with central venous catheters (CVCs) due to species that are known skin colonizers, such as *C. auris* and *C. parapsilosis*. BDG was not helpful in anticipating the diagnosis of candidemia, but, on the contrary, higher levels of serum BDG were detected after the onset of invasive infection. This might be explained by a sudden inoculation of high fungal load through colonized or infected CVCs, in contrast to the progressive increase in fungal burden released in the bloodstream in the case of abdominal origin of candidemia. Unfortunately, this hypothesis could not be confirmed by our data as exact time-to-positivity for positive blood cultures drawn from CVC and peripheral vein, CVC tip cultures, and timing of CVC removal were not available for all the included subjects. An important aspect to be considered is that a reduced sensitivity may cast further doubts on the usefulness of BDG in hospitals where certain non-*albicans* species are more prevalent. In this regard, we think an interesting research question is to assess whether combinations of diagnostic biomarkers could improve the diagnostic accuracy of BDG in similar scenarios [18,19,20].

Limitations of our study are its retrospective nature and the predominance of *C. parapsilosis* infections, although the sample included is representative of the overall epidemiology of candidemia in our ICUs. Only 21 episodes of *C. auris* candidemia were included, and this might have impaired the power of our observation in detecting a difference in sensitivity of serum BDG. Moreover, repeated serum BDG values were available only for 1 in 4 episodes, thus impairing our ability to clearly evaluate the trend of BDG values over time. Some potential additional causes of high BDG levels (e.g., the administration of intravenous immunoglobulins) in the days preceding candidemia might have occurred, likely contributing to an increase in the observed sensitivity of BDG. Finally, as mentioned above, the lack of complete data on the rate of CVC-related infections and their management is a limitation in interpreting the findings of our study.

In conclusion, in critically ill patients admitted to the ICU, serum BDG levels were lower in *C. parapsilosis* and *C. auris* candidemia compared to episodes caused by *C. albicans*, with an overall rather low sensitivity of BDG for the diagnosis of bloodstream infections. Local epidemiology, with particular attention to emerging non-*albicans* species of *Candida*, should be carefully considered when assessing the role of BDG in the diagnosis and management of empirical therapy in ICU patients with suspected candidemia.

## Figures and Tables

**Table 1:** Serum BDG values, overall sensitivity, and sensitivity stratified according to *Candida* species.

*Candida* Species (Total Number of Episodes = 146; Total Number of BDG Samples = 187) ^§^	Median BDG Value (IQR), in pg/mL ^§§^	Median Time from Candidemia Onset to First BDG Determination (IQR), in Days	Sensitivity (95% CI)
For all samples (*n* = 187)	84 (21–314)	−0.06 (−1.28, 0.83)	51.3% (44.1–58.5%)
For all episodes (*n* = 146)			47.3% (39.0–55.0%)
For *C. albicans* (*n* = 40) samples	182 (30.5–523)	0 (−1.16–0.78)	65.0% (48.7–78.4%)
For *C. albicans* (*n* = 29) episodes			62.1% (42.8–78.2%)
For *C. parapsilosis* (*n* = 105) samples	78 (19–290)	−0.08 (−1.29–1.00)	48.6% (39.1–58.2%)
For *C. parapsilosis* (*n* = 84) episodes			44.0% (33.7–54.9%)
For *C. auris* (*n* = 26) samples	48 (15–159)	0 (−1.99–0.83)	42.3% (24.5–62.4%)
For *C. auris* (*n* = 21) episodes			42.9% (23.0–65.3%)
For other species (*n* = 16) samples *	81 (29–195)	−0.62 (−1.99–0.53)	50.0% (25.6–74.4%)
For other species (*n* = 12) episodes **			41.7% (16.4–72.2%)

^§^ Number of episodes refers to the number of candidemia episodes; ^§§^ Referred to total number of BDG samples; * *C. glabrata n* = 11, *C. tropicalis n* = 4, *C. lusitaniae n* = 1; ** *C. glabrata n* = 7, *C. tropicalis n* = 4, *C. lusitaniae n* = 1; BDG: b-D-glucan, 95% CI: 95% confidence interval; IQR: interquartile range.

**Table 2:** Description of serum BDG concordance/discordance in 37 candidemia episodes with ≥2 BDG results available.

BDG Values *	All (*n* = 37)	*C. albicans* (*n* = 10)	*C. auris* (*n* = 5)	*C. parapsilosis* (*n* = 18)	Other Species (*n* = 4)
**Discordant values**	7 (18.9)	1 (10)	2 (40)	3 (16.7)	1 (25)
**Concordant values**	30 (81.1)	9 (90)	3 (60)	15 (83.3)	3 (75)

* Concordant values are defined as 2 or more serum BDG results all either negative (<80 pg/mL) or positive (≥80 pg/mL), while discordant values are defined as 2 values belonging to different categories: positive and negative. No differences were observed between groups: *C. albicans* vs. *C. auris* (*p* = 0.24), *C. albicans* vs. *C. parapsilosis* (*p* = 0.99), *C. auris* vs. *C. parapsilosis* (*p* = 0.29). BDG: b-D-glucan, 95% CI: 95% confidence interval; IQR: interquartile range.

## Data Availability

The data that support the findings of this study are available from the corresponding author upon reasonable request.

## Supplementary Materials

The following supporting information can be downloaded at: https://www.mdpi.com/article/10.3390/jof8090921/s1, Figure S1: Trend over time of serum BDG values stratified according to *Candida* species causing candidemia, when two or more samples were available.

## References

[1] Patterson T.F., Donnelly J.P. (2019). New Concepts in Diagnostics for Invasive Mycoses: Non-Culture-Based Methodologies. J. Fungi.

[2] Karageorgopoulos D.E., Vouloumanou E.K., Ntziora F., Michalopoulos A., Rafailidis P.I., Falagas M.E. (2011). β-D-Glucan Assay for the Diagnosis of Invasive Fungal Infections: A Meta-Analysis. Clin. Infect. Dis..

[3] Rouzé A., Estella Á., Timsit J.-F. (2022). Is (1,3)-β-d-Glucan Useless to Guide Antifungal Therapy in ICU?. Intensive Care Med..

[4] Martín-Mazuelos E., Loza A., Castro C., Macías D., Zakariya I., Saavedra P., Ruiz-Santana S., Marín E., León C. (2015). β-d-Glucan and Candida Albicans Germ Tube Antibody in ICU Patients with Invasive Candidiasis. Intensive Care Med..

[5] Lamoth F., Cruciani M., Mengoli C., Castagnola E., Lortholary O., Richardson M., Marchetti O. (2012). Third European Conference on Infections in Leukemia (ECIL-3) β-Glucan Antigenemia Assay for the Diagnosis of Invasive Fungal Infections in Patients with Hematological Malignancies: A Systematic Review and Meta-Analysis of Cohort Studies from the Third European Conference on Infections in Leukemia (ECIL-3). Clin. Infect. Dis..

[6] Mikulska M., Balletto E., Castagnola E., Mularoni A. (2021). Beta-D-Glucan in Patients with Haematological Malignancies. J. Fungi.

[7] Bloos F., Held J., Kluge S., Simon P., Kogelmann K., de Heer G., Kuhn S.-O., Jarczak D., Motsch J., Hempel G. (2022). (1 → 3)-β-d-Glucan-Guided Antifungal Therapy in Adults with Sepsis: The CandiSep Randomized Clinical Trial. Intensive Care Med..

[8] León C., Ruiz-Santana S., Saavedra P., Castro C., Loza A., Zakariya I., Úbeda A., Parra M., Macías D., Tomás J.I. (2016). Contribution of Candida Biomarkers and DNA Detection for the Diagnosis of Invasive Candidiasis in ICU Patients with Severe Abdominal Conditions. Crit. Care.

[9] Cornely O.A., Bassetti M., Calandra T., Garbino J., Kullberg B.J., Lortholary O., Meersseman W., Akova M., Arendrup M.C., Arikan-Akdagli S. (2012). ESCMID* Guideline for the Diagnosis and Management of Candida Diseases 2012: Non-Neutropenic Adult Patients. Clin. Microbiol. Infect..

[10] Martin-Loeches I., Antonelli M., Cuenca-Estrella M., Dimopoulos G., Einav S., De Waele J.J., Garnacho-Montero J., Kanj S.S., Machado F.R., Montravers P. (2019). ESICM/ESCMID Task Force on Practical Management of Invasive Candidiasis in Critically Ill Patients. Intensive Care Med..

[11] Mikulska M., Giacobbe D.R., Furfaro E., Mesini A., Marchese A., Del Bono V., Viscoli C. (2016). Lower Sensitivity of Serum (1,3)-β-d-Glucan for the Diagnosis of Candidaemia Due to Candida Parapsilosis. Clin. Microbiol. Infect..

[12] Chibabhai V., Fadana V., Bosman N., Nana T. (2019). Comparative Sensitivity of 1,3 Beta-D-Glucan for Common Causes of Candidaemia in South Africa. Mycoses.

[13] Farooqi J., Niamatullah H., Irfan S., Zafar A., Malik F., Jabeen K. (2021). Comparison of β-d-Glucan Levels between Candida Auris and Other Candida Species at the Time of Candidaemia: A Retrospective Study. Clin. Microbiol. Infect..

[14] Mikulska M., Furfaro E., Magnasco L., Codda G., Giacobbe D.R., Dentone C., Vena A., Marchese A., Bassetti M. (2022). Levels of Beta-D-Glucan in Candida Auris Supernatants, an in Vitro and in Vivo Preliminary Study. Clin. Microbiol. Infect..

[15] Kritikos A., Poissy J., Croxatto A., Bochud P.-Y., Pagani J.-L., Lamoth F. (2020). Impact of the Beta-Glucan Test on Management of Intensive Care Unit Patients at Risk for Invasive Candidiasis. J. Clin. Microbiol..

[16] Haydour Q., Hage C.A., Carmona E.M., Epelbaum O., Evans S.E., Gabe L.M., Knox K.S., Kolls J.K., Wengenack N.L., Prokop L.J. (2019). Diagnosis of Fungal Infections. A Systematic Review and Meta-Analysis Supporting American Thoracic Society Practice Guideline. Ann. Am. Thorac. Soc..

[17] De Pascale G., Posteraro B., D’Arrigo S., Spinazzola G., Gaspari R., Bello G., Montini L.M., Cutuli S.L., Grieco D.L., Di Gravio V. (2020). (1,3)-β-d-Glucan-Based Empirical Antifungal Interruption in Suspected Invasive Candidiasis: A Randomized Trial. Crit. Care.

[18] Giacobbe D.R., Asperges E., Cortegiani A., Grecchi C., Rebuffi C., Zuccaro V., Scudeller L., Bassetti M., The FUNDICU investigators (2022). Performance of Existing Clinical Scores and Laboratory Tests for the Diagnosis of Invasive Candidiasis in Critically Ill, Nonneutropenic, Adult Patients: A Systematic Review with Qualitative Evidence Synthesis. Mycoses.

[19] Giacobbe D.R., Mikulska M., Tumbarello M., Furfaro E., Spadaro M., Losito A.R., Mesini A., De Pascale G., Marchese A., Bruzzone M. (2017). Combined Use of Serum (1,3)-β-d-Glucan and Procalcitonin for the Early Differential Diagnosis between Candidaemia and Bacteraemia in Intensive Care Units. Crit. Care.

[20] Martínez-Jiménez M.C., Muñoz P., Valerio M., Alonso R., Martos C., Guinea J., Bouza E. (2015). Candida Biomarkers in Patients with Candidaemia and Bacteraemia. J. Antimicrob. Chemother..

