# Supplementary material for: Sensitivity of Serum Beta-D-Glucan in Candidemia According to Candida Species Epidemiology in Critically Ill Patients Admitted to the Intensive Care Unit
Source: J Fungi (Basel). 2022 Aug 30;8(9):921. doi: 10.3390/jof8090921 (PMC9503671; doi:10.3390/jof8090921)

Supplementary Figure S1. Trend over time of serum BDG values stratified according to *Candida* species causing candidemia, when two or more samples were available (total n= 37).

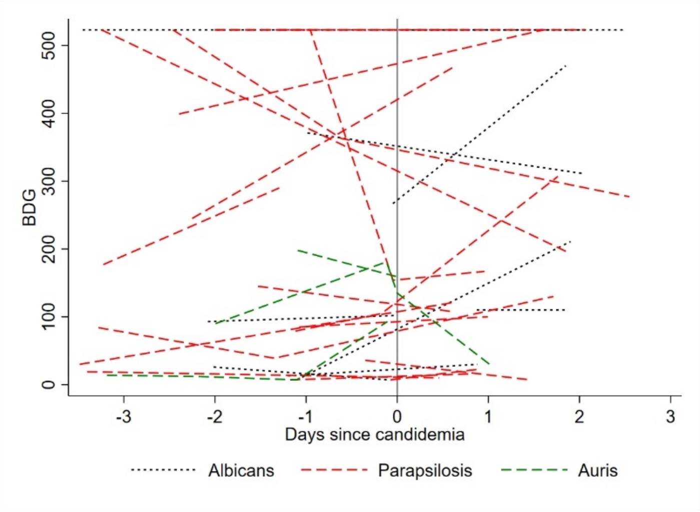

Supplement: Supplementary file 1 [file jof-08-00921-s001.zip › jof-1864272-supplementary.pdf]
